# Serum Uric Acid Is Independently Associated with Coronary Calcification in an Asymptomatic Population

**DOI:** 10.1007/s12265-018-9843-8

**Published:** 2018-11-09

**Authors:** Loretta Zsuzsa Kiss, Zsolt Bagyura, Csaba Csobay-Novák, Árpád Lux, Lívia Polgár, Ádám Jermendy, Pál Soós, Zsolt Szelid, Pál Maurovich-Horvat, Dávid Becker, Béla Merkely

**Affiliations:** 1 Heart and Vascular Center, Semmelweis University, Városmajor Street 68, Budapest, H-1122 Hungary; 2 MTA-SE Cardiovascular Imaging Research Group, Heart and Vascular Center, Semmelweis University, Városmajor Street 68, Budapest, H-1122 Hungary

**Keywords:** Cardiovascular screening, Hyperuricemia, Coronary artery calcium score

## Abstract

Detecting early-stage atherosclerosis is an important step towards cardiovascular disease prevention. Coronary artery calcium (CAC) score is a sensitive and non-invasive tool for detecting coronary atherosclerosis. Higher serum uric acid (SUA) levels are known to be associated with cardiovascular diseases. However, there is inconsistency regarding the independence of the association. The aim of our study was to assess the association of CAC and SUA in an asymptomatic population. CAC scans of 281 participants were analyzed in a voluntary screening program. A health questionnaire, physical examination, and laboratory tests were also performed. Participants with a history of cardiovascular disease were excluded from the analysis. 36.3% (*n* = 102) of the participants had no detectable CAC and 13.9% (*n* = 39) had a CAC score of > 300. SUA showed positive correlation with CAC score (0.175, *p* < 0.01). SUA was independently associated with Ca score > 300 (OR 5.17, *p* = 0.01) after the effects of conventional risk factors were eliminated.

## Introduction

Atherosclerosis is a systemic disease and it is among the leading causes of mortality [1]. Its local manifestation in the heart is coronary artery disease. The developing plaque causes increasing stenosis, which may result in life-threatening clinical manifestations, such as acute coronary syndrome (ACS) (unstable angina, acute myocardial infarction). Detecting atherosclerosis in subclinical stages is an important part of personalized cardiovascular risk assessment and therefore the basis of prevention.

Low-dose computer tomography (CT) scanning of the heart is a non-invasive method that provides quantitative information of the calcification of the coronary vessels. The extent of calcification is measured in Agatston score, which shows a strong correlation with the total plaque burden and therefore provides information about the severity of the coronary atherosclerosis [2]. Coronary calcium score is a strong predictor of cardiac events and it refines cardiovascular risk assessment beyond conventional risk factors [3].

Uric acid is the final breakdown product of purine metabolism via an enzymatic reaction involving xanthine oxidase and is excreted in urine [4]. Plasma level of urate depends on the balance between the amount of purine intake with food, the amount of urate synthesized, and the amount that is excreted in urine or through the gastrointestinal tract. Increased production or decreased excretion of uric acid may lead to hyperuricemia [5]. Men have higher levels than women before menopause [6]. High serum uric acid (SUA) level is known to be associated with various CV risk factors and diseases such as hypertension, diabetes mellitus, and metabolic syndrome. Recently, Degli Esposti et al. showed that higher SUA levels are linked with an increased risk of hospitalizations related to chronic kidney diseases, mortality, and also CVDs [7]. Its role as a risk factor for cardiovascular disease is studied extensively; however, whether the association is independent of conventional risk factors is controversial.

In the present study, our aim was to assess the prevalence of subclinical atherosclerosis and its associations with SUA and conventional risk factors in an asymptomatic population. Identification of an association between SUA levels and CACS may have significant implications for screening patients for atherosclerotic coronary artery disease (CAD).

## Methods

A cross-sectional voluntary cardiovascular screening program (Budakalász Health Survey) was performed, in 2011–2013. The target population was the adult population (> 20 years, ~ 8000 inhabitants) of the selected central Hungarian town; participation rate was around 30%. The screening program had two main parts. The Health Interview Survey is based on the European Health Interview Survey with added questions on cardiovascular diseases. Questions cover the following topics: socio-economic status, health status, health determinants. Medical history of participants was recorded by a physician with special attention to cardiovascular diseases, related signs and symptoms, lifestyle (alcohol consumption, sport activities, smoking habits), and family history. Anthropometric data was also collected (such as body height, weight, waist circumference). Venous blood was taken for laboratory tests and biobanking. All laboratory tests were performed in central laboratory and included blood cell count, renal function, hepatic enzymes, electrolytes, albumin, glucose, LDL-, HDL-cholesterol, TG, uric acid, hsCRP, and HbA1c%. Concentration of SUA was measured by using a colorimetric assay with rigorous quality control (Roche Diagnostics Ltd., Mannheim, Germany).

A CAC scan (Brilliance iCT, Philips Healthcare, Best, The Netherlands) was performed in men older than 35 years and women older than 40 years on a voluntary base. Radiation dose was 0.5 mSv or less. Oral beta-blockers were administered, if the heart rate was above 65 bpm. Prospectively, ECG-triggered scans were acquired. Quantitative analysis of coronary calcification on the axial images was performed using a commercially available software application (Calcium scoring, Heartbeat-CS, Philips Healthcare). All coronary artery plaques with an area of ≥ 1 mm^2^ and a density of greater than 130 Hounsfield Units (HU) were identified by the software, and then real coronary plaques were selected manually by an expert observer which allow the semiautomatic software to calculate the Agatston score, calcification area and volume. The 0 value of Agatston score has a negative predictive value of nearly 100% for ruling out obstructive CAD [8] and indicates a very low CV event rate. CAC level of 300 or more is associated with elevated risk for coronary heart disease even in low- and intermediate-risk persons [9, 10]; therefore, we used 0 and 300 as cutoff values in the analyses.

Medical history was regarded positive for hypertension, hyperlipidaemia, and diabetes mellitus if it was formerly diagnosed or the patient received treatment. Body mass index was calculated by the Quetelet’s formula. Blood pressure measurement was performed on the arms after 20 min rest in a supine position. Blood pressure above 140 mmHg systolic and/or 90 mmHg diastolic was defined as pathologically high.

Total number of 511 participants volunteered for CT scan between 2011 and 2013, 41.1% of them were male. Participants with the following cardiovascular history were excluded from the examination: 24 patients with previous myocardial infarction (4.7%), 95 patients with angina pectoris (18.6%), 10 patients with percutaneous coronary intervention (PCI) (2.0%), 5 patients with coronary artery bypass graft (1.0%), 48 patients with known heart failure (9.2%), 4 patients with cardiomyopathy (0.8%), 20 patients with stroke (3.9%), 4 patients with transient ischemic attack (0.8%), and 38 patients with peripheral artery disease (7.5%). Also, those on regular allopurinol therapy (17, 3.3%) were excluded from analysis. As a patient could have more than one exclusion criteria, overall 281 participants were finally included in the analysis, 41.3% of them male.

Power analysis was conducted using an online calculator developed by MGH Biostatistics Center.^1^ Data used in the power analysis: *N* = 281, *α* = < 0.05, s1 = 312.9 (dependent variable, Ca score), s2 = 81.9 (independent variable, SUA), the minimal detectable difference entered was 0.691. The analysis revealed that there is an 85% probability of detecting a relationship between the independent and the dependent variables in this study.

### Statistical Methods

Microsoft Excel and PASW Statistics 18 (SPSS) were used for statistical analysis. All continuous variables were expressed as mean with standard deviation (SD) or as medians with interquartile range as appropriate depending on the distribution of the values, whereas categorical variables were expressed as percentage. Comparisons of means, medians, and proportions were performed with variance analysis, Kruskal-Wallis test, Jonckheere-Terpstra test, chi-square tests, and Cochran-Armitage test respectively. Spearman correlation was used to test the association between SUA and CAC. Multivariate regression analysis was performed adjusted for age, gender, and risk factors with the 1st SUA tercile as reference category. All analyses were performed two-tailed and *p* < 0.05 was considered significant.

## Results

The mean age was 60 (± 10.9) years, the average BMI was 27.87 (±4.96), average Framingham 10-year risk of developing cardiovascular disease score was 17.6 (±12.2), and all patients belonged to the Caucasian race. Mean serum uric acid level was 355.2 ± 87.5 μmol/L in men and 269.6 ± 65.5 μmol/L in women. As SUA level differs substantially between males and females [11], therefore, based on the distribution, the SUA level was stratified into terciles by gender. Characteristics of serum uric acid in the three terciles groups in males and females are shown in Table 1.

**Table 1:** Terciles of serum uric acid by gender in the study population

Tercile	Males	Females
1	0–315.3	0–251.9
2	315.3–382.6	252–310.9
3	382.7–	311–

Serum uric acid is given in μmol/l

Age, BMI, hypertension, HDL, triglyceride, and creatinine levels were significantly different in the three tercile groups. In the 3rd SUA tercile group, age, BMI, and creatinine level were higher, whereas HDL was lower compared to the other groups. Also, the frequency of hypertension increased gradually in the groups and was highest in the third. Triglyceride level and SBP were lowest in group 1, and similarly high in groups 2 and 3. There was no significant difference in the gender distribution among the three groups. Clinical baseline characteristics of the 281 patients are described in Table 2.

**Table 2:** Clinical characteristics in the SUA terciles

	SUA tercile 1 (*n* = 93)	SUA tercile 2 (*n* = 94)	SUA tercile 3 (*n* = 94)	Total (*n* = 281)	*p*
Age, mean (SD)	57.22 (11.67)	60.54 (11.21)	62.30 (9.10)	60.03 (10.89)	0.005
Gender, male, *n* (%)	38 (40.9%)	39 (41.5%)	39 (41.5%)	116 (41.3%)	0.995
BMI, mean (SD)	25.81 (3.92)	28.23 (5.19)	29.55 (4.96)	27.87 (4.96)	< 0.001
Hypertension, *n* (%)	32 (34.4%)	47 (50.0%)	63 (67.0%)	142 (50.5%)	< 0.001
Hyperlipidaemia, *n* (%)	36 (38.7%)	35 (37.2%)	42 (42.7%)	113 (40.1%)	0.545
DM, *n* (%)	7 (7.5%)	13 (13.8%)	8 (8.5%)	28 (10.0%)	0.301
Active smoker, *n* (%)	17 (18.3%)	10 (11.6%)	7 (7.4%)	34 (12.1%)	0.066
SBP, mean (SD)	132.8 (16)	138.9 (18)	137.1 (17.0)	136.3 (17.1)	0.046
DBP, mean (SD)	79.3 (9.1)	81 (8.9)	80.6 (9.9)	80.3 (9.3)	0.214
LDL, mean (SD)	3.39 (1.00)	3.54 (0.98)	3.62 (1.01)	3.52 (0.99)	0.269
HDL, mean (SD)	1.65 (0.49)	1.53 (0.49)	1.49 (0.46)	1.55 (0.49)	0.049
Triglyceride, mean (SD)	1.97 (1.31)	2.49 (1.56)	2.48 (1.39)	2.31 (1.44)	0.019
Total cholesterol, mean (SD)	5.59 (1.14)	5.79 (1.07)	5.84 (1.16)	5.74 (1.13)	0.287
Serum creatinine, mean (SD)	73.45 (14.01)	74.41 (15.28)	81.50 (17.56)	76.47 (16.04)	0.001
Serum uric acid, mean (SD)	239.86 (367.50)	308.24 (37.70)	401.10 (65.82)	317.67 (81.90)	< 0.001

### CT Scan Results

Total Agatston score was 0 in 102 cases (36.3%) and above 300 in 39 cases (13.9%). Median total Ca score was 14.34 (IQR 0–107.4). The Jonckheere-Terpstra test for total Ca score confirmed a trend across SUA groups (J-T statistic 13160 ± 724, *p* < 0.001). Serum uric acid level showed a positive correlation with total coronary calcium score (*r* = 0.281, *p* < 0.001). Frequencies of Ca score > 0, and > 300 was significantly different in the three SUA groups (Table 3). Also, there was a trend across SUA groups in both cases (CA statistic 9.59, *p* = 0.002 and 8.597, *p* = 0.003, respectively.)

**Table 3:** Median total coronary calcium scores and frequencies of Ca score > 0, and > 300 in the SUA terciles

	SUA tercile 1	SUA tercile 2	SUA tercile 3	Total	*p*
Total Ca score, median (IQR)	0.7 (0–49.05)	19.37 (0–159.33)	33.74 (0–156.58)	14.34 (0–107.4)	0.001
Ca score > 0, *n* (%)	49 (52.7%)	60 (63.8%)	70 (74.5%)	179 (63.7%)	0.008
Ca score > 300, *n* (%)	5 (5.4%)	15 (16.0%)	19 (20.2%)	39 (13.9%)	0.01

### Multivariate Analysis

We performed multivariate adjustment for conventional cardiovascular risk factors (gender, age, BMI, hypertension, hyperlipidaemia, diabetes mellitus, smoking status, creatinine, and SUA terciles as covariates). In model 1, the dependent variable was the presence of any coronary calcification (Agatston score > 0), and in model 2, the presence of coronary calcification indicating high CV risk (Agatston score > 300). Multivariate logistic regression analysis revealed that in model 1, SUA was not an independent predictor of the presence of overall (Agatston score > 0) coronary calcification (Table 4). In model 2, compared to the lowest SUA tercile (reference category), the third SUA tercile is an independent predictor (OR 5.17, *p* = 0.010) for the presence of severe significant coronary calcification (Ca score > 300). In this model, male gender (OR 3.041, p = 0,017), age (OR 1.13, *p* < 0.001), active smoking (OR 7.152, *p* = 0.004), and history of hypertension (3.336, *p* = 0.022) were independently associated with coronary calcification.

**Table 4:** Results of the logistic regression analysis: age, gender, and risk factor-adjusted odds ratios with 95% confidence intervals for the presence of Ca score > 0 and Ca score > 300 for SUA terciles

	Model 1^f^ - Ca score > 0				Model 2^g^ - Ca score ≥ 300			
	OR	*p*	95% CI		OR	*p*	95% CI	
			Lower	Upper			Lower	Upper
Gender (male)	2.39	0.01	1.22	4.71	3.04	0.02	1.22	7.61
BMI^a^	1.00	0.97	0.94	1.06	1.03	0.54	0.95	1.11
Age	1.08	< 0.001	1.08	1.11	1.13	< 0.001	1.07	1.20
DM^b^	1.40	0.59	0.42	4.36	1.70	0.31	0.61	4.78
SUA^c^ reference		0.56				0.04		
SUA tercile 2	1.23	0.56	0.62	2.42	3.21	0.06	0.93	11.04
SUA tercile 3	1.51	0.28	0.72	3.17	5.17	0.01	1.48	18.03
Creatinine	1.01	0.21	0.99	1.04	0.99	0.27	0.96	1.01
Smoking	1.08	0.87	0.47	2.48	7.15	0.00	1.86	27.57
HT^d^	1.85	0.05	1.00	3.45	3.34	0.02	1.19	9.36
HLP^e^	1.19	0.55	0.67	2.12	1.62	0.24	0.72	3.62

^a^Body mass index

^b^Diabetes mellitus

^c^Serum uric acid

^d^Hypertension

^e^hyperlipidaemia

^f^Model 1 R square –Cox&Snell: 0.212, Nagelkerke 0.29

^g^Model 2 R square –Cox&Snell: 0.194, Nagelkerke 0.35

## Discussion

In our study, we presented an independent association of serum uric acid level with severe coronary calcification in an asymptomatic population. We found that serum uric acid level showed a positive correlation with total coronary calcium score. Compared to the lowest SUA tercile, the third SUA tercile was an independent predictor for the presence of high-risk coronary calcification. Moreover, gender, age, smoking, and history of hypertension were independently associated with coronary calcification.

Levels of serum uric acid in our study population were similar to those in other central European population studies [12]. In the groups based on SUA terciles age, history of hypertension, BMI, HDL, triglyceride, serum creatinine levels, and Agatston score showed an increasing tendency, with higher values in the third tercile group. It is known that SUA level gradually increases with age and that it is associated with obesity [13, 14]. An analysis from the Framingham Heart Study found a positive association of higher SUA levels and an increase in both systolic (SBP) and diastolic blood pressure (DBP) [15]. In our study population SBP but not DBP was significantly different in the three SUA terciles. We found a difference in HDL and triglyceride levels but not in history of hyperlipidaemia, LDL or total cholesterol levels between the groups that may suggest that metabolic changes linked with increasing SUA level affect HDL levels more. These findings are in line with other authors’ results [16, 17].

Serum creatinine showed significantly higher levels across SUA tercile groups. It is known that higher serum creatinine levels could be a result of attenuated kidney function that may cause higher serum uric acid levels. According to our results, a higher SUA level is associated with severe coronary calcification independently from serum creatinine levels and therefore renal function.

Several studies have demonstrated a relationship between coronary calcification and SUA levels [18, 19], but other studies do not support these findings [20, 21]. Krishnan et al. [18] found that Agatston score (> 0) is associated with SUA levels in young healthy adults. In their analyses, in contrast to our results, age was not significantly associated with SUA concentration, and approximately 40% of the population was African-American. Our study population is older, central European and Caucasian.

Grossman et al. [22] demonstrated that high serum uric acid levels are associated with Agatston score (> 0) in a predominantly male study population. They found that patients in the highest SUA tertile were younger and more likely male that is in contrast to our results. The reason behind that may be that in our study the gender ratio was more balanced and SUA level was stratified into tertiles by gender as SUA level differs substantially between males and females [11]. Moreover, patients on allopurinol treatment were excluded from the analysis. In contrast to the abovementioned studies, we analyzed not only the existence of overall coronary calcification (Agatston > 0), but we also used the cutoff value of 300 for Agatston score that defines high-risk patient group and found a strong and independent association.

It is known that SUA is associated with the progression of coronary calcification. Based on the findings of Bjornstad et al., in a study with 6-year follow-up time SUA predicts the progression of calcium score in patients with type 1 diabetes mellitus [23]. Calvo et al. had concordant results in a study investigating postmenopausal women [24]. In contrast to our results these authors did not find an association between SUA levels and CAC severity.

Of course, we are not yet sure if SUA could have a direct and independent pathogenetic effect on subclinical atherosclerosis, and especially on coronary calcification. Sharaf et al. described in a review that the role of SUA in CV disease is still contradictory [25]. SUA could be a risk marker, a risk factor, or it is possible that lowering SUA could improve the outcome of CV diseases [26]. The most frequent confounding factors are HT, dyslipidemia and DM. The presence of these and other factors that are even harder to assess (for example alcohol consumption, anti-hypertensive treatment, diet) and the diversity of the studied populations may be the cause of the contradicting results. A review by Feig et al. from 2008 concludes that the effect of higher serum uric acid levels on atherosclerosis is not independent of hypertension; moreover, they suggest an indirect effect as SUA may acts through inducing high blood pressure [27]. Reschke et al. in a prospective observational study of hypertensive children concluded that UA is not an independent CVD risk factor rather a marker of obesity [28]. Li et al. found an association between hyperuricemia and subclinical atherosclerosis in a Chinese population that was only independent in men [29]. Recent evidences are in favor of the independence of the association. Li et al. found an independent association between SUA levels and the prevalence of vulnerable carotid plaque in the APAC study [30].

A strong direct association is plausible between SUA and both structural and functional changes of arteries according to several studies. There is evidence that uric acid may play a causal role in the development of endothelial dysfunction and atherosclerosis [31]. It seems that uric acid has direct effects on impaired nitrogen oxide (NO) production and stimulating vascular smooth muscle cell (VSMC) proliferation causing endothelial dysfunction. Endothelial xanthine-oxidase (XO) contributes to vascular damage via reactive oxygen species (ROS) production (during the conversion of hypoxanthine into xanthine and then uric acid), and XO in macrophages upregulates foam cell formation by increasing the uptake of modified low-density lipoprotein (LDL) or very low-density lipoprotein (VLDL). Moreover, uric acid may cause endothelial dysfunction directly by impaired nitrogen oxide production and stimulation of VSMC proliferation [32]. Indirectly, uric acid may cause metabolism-induced inflammation through inflammasome activation by crystallized uric acid particles or superoxide free radicals generated by xanthine-oxidase (XO) [33].

These alterations could somehow represent the pathophysiological bridge between SUA and atherosclerosis in the coronary arteries. For developing a sufficient prevention strategy, it is substantial to hold thorough knowledge of the prevalence, extent, and variability of different risk factors. Accordingly, the impact of urate lowering treatments on preventing coronary calcification should be further investigated. And also, we must take into consideration the specific attributes, needs, and inclinations of the target population.

Our study has some limitations. As the participation to the study is on voluntary basis, there is the possibility that subjects with known hypertension or other factors associated with coronary calcification had a greater propensity to participate. Secondly, a relatively low number of asymptomatic patients are included in our study, as we indicated low-dose CT only for men above 35 and women above 40; therefore, patients with lowest cardiovascular risk are excluded from our study.

In conclusion, in an asymptomatic population, SUA levels were associated with calcium scores and the third SUA tercile was an independent predictor for the presence of high-risk coronary calcification. Moreover, gender, age, smoking, and history of hypertension were independently associated with severe coronary calcification.

## Abbreviations

ACS: Acute coronary syndrome
BMI: Body mass index
CAC: Coronary artery calcium
CAD: Coronary artery disease
CI: Confidence interval
CT: Computer tomography
CV: Cardiovascular
DBP: Diastolic blood pressure
DM: Diabetes mellitus
HDL: High-density lipoprotein
HT: Hypertension
HU: Hounsfield Unit
LDL: Low-density lipoprotein
OR: Odds ratio
PCI: Percutaneous coronary intervention
ROS: Reactive oxygen species
SBP: Systolic blood pressure
SD: Standard deviation
SUA: Serum uric acid
TG: Triglyceride
XO: Xanthine-oxidase

## Footnotes

1
http://hedwig.mgh.harvard.edu/sample_size/js/js_associative_quant.html
